# Two mouse lines selected for large litter size display different lifetime fecundities

**DOI:** 10.1530/REP-20-0563

**Published:** 2021-04-20

**Authors:** Martina Langhammer, Erika Wytrwat, Marten Michaelis, Jennifer Schön, Armin Tuchscherer, Norbert Reinsch, Joachim M Weitzel

**Affiliations:** 1 Institut für Fortpflanzungsbiologie, Leibniz-Institut für Nutztierbiologie (FBN), Dummerstorf, Germany; 2 Institut für Genetik und Biometrie, Leibniz-Institut für Nutztierbiologie (FBN), Dummerstorf, Germany

## Abstract

We recently described two outbred mouse lines that were selected for large litter size at first delivery. However, lifetime fecundity appears to be economically more important for the husbandry of many polytocous species for which mouse lines might serve as bona fide animal models (e.g. for pigs). In the present study, we compared the lifetime fecundities of two highly fertile mouse lines (FL1 and FL2: >20 offspring/litter at first delivery) with those of an unselected control line (ctrl) and two lines that were selected for high body weight (DU6) and high protein mass (DU6P) without selection pressure on fertility. We tested the hypothesis that selection for large litter size at first parturition would also increase lifetime fecundity in mice, and we observed very large differences between lines. Whereas FL1 and ctrl delivered up to nine and ten litters, none of the DU6 and DU6P females gave birth to more than five litters. In line with this observation, FL1 delivered the most pups per lifetime (85.7/female). FL2 females produced the largest average litter sizes (20.4 pups/litter) in the first four litters; however, they displayed a reduced number of litters. With the exception of ctrl, litter sizes declined from litter to litter. Repeated delivery of litters with high offspring numbers did not affect the general health of FL females. The presented data demonstrate that two biodiverse, highly fertile mouse lines selected for large litter size at first delivery show different lifetime reproductive fitness levels. Thus, these mouse lines might serve as valuable mouse models for investigating lifetime productivity and longevity in farm animals.

## Introduction

Fertility research takes advantage of using informative animal models. Worldwide, more than 1000 mouse models show a specific fertility phenotype (Matzuk & Lamb 2008, Jamsai & O’Bryan 2011, Ogorevcet al. 2011). Among them, approximately 99% show a decreased fertility phenotype. A fertility phenotype causing sub or infertility is easily detectable in a standard breeding protocol if a novel mouse model has been developed, even if the focus of the research is not primarily addressing fertility questions. In contrast, fertility phenotypes associated with increased fertility are not as easily detectable, as subtle increases in litter size could easily be overlooked. Therefore, only ~1% of mouse models showing a fertility phenotype are associated with the annotations ‘enhanced fertility’ and/or ‘increased litter size’ (Mouse Genome Informatics, MGI – www.informatics.jax.org).

Mouse models showing an increased fertility phenotype can be divided into two groups: (i) ‘classical’ transgenic or knockout inbred models and (ii) outbred mouse models. Only approximately a dozen transgenic or knockout inbred mouse models show an increased fertility phenotype. One example is the Bcl-2 knockout mouse. Due to the genetic inactivation of the Bcl-2 gene, affected females show decreased ovarian somatic cell apoptosis followed by enhanced folliculogenesis and a higher ovulation rate (Hsu* et al.* 1996, Hutt 2015). A higher ovulation rate due to an accelerated reproductive cycle has also been described for Gpr149-knockout females (Edson* et al.* 2010). However, it should be noted that physiological consequences are moderate in these animal models. Typically, enhanced litter sizes of 10–20% have been reported.

In contrast to classical inbred mouse models, several attempts have been made to select for higher fertility through selective breeding, for example, in pigs and rabbits (Johnson* et al.* 1999, Su* et al.* 2007, Ziadi* et al.* 2013). Two groups consequently developed outbred mouse models over more than 100 generations of selection. The Odd Vangen group at the Agricultural University of Norway in Ås developed an outbred mouse model that has been selected for high fertility (Holt* et al.* 2004, 2005). These animals almost doubled the number of offspring per litter to 21.6 pups per litter (Holt* et al.* 2004, 2005). Unfortunately, this line was terminated in 2007 after 130 generations of selection; however, based on this genetic resource, the inbred line QSi5 was developed, which maintained an elevated litter size (Wei* et al.* 2013). In addition to the Norwegian line and its branch, two high-fertility outbred mouse models have been established at the FBN Dummerstorf (Leibniz Institute for Farm Animal Biology (FBN), Dummerstorf).

At the FBN Dummerstorf, the two high-fertility mouse lines (fertility lines 1 and 2, FL1 and FL2) were developed by creating the unselected control line FZTDU (ctrl) as the initial population after crossing four inbred and four outbred founder mouse lines (Dietl* et al.* 2004) and maintaining two long-term outbred selection lines since the 1970s. In each new generation, animals were selected according to a selection criterion that includes both (i) the number of live pups per litter and ii) litter birth weight. Animals born from the largest and heaviest litters were then chosen as parents of the next generation (Schüler & Bünger 1982). To keep the generation interval short, each dam only had a single litter, and consequently, the selection was solely on litter size at the first parturition. After >45 years and 181 generations of selection on the trait ‘litter size at first delivery’, these mouse lines almost doubled the number of offspring per litter (to 20.6 and 21.4 pups per litter) in comparison to an unselected control line originating from the same founder population (11.5 pups per litter) (Michaelis* et al.* 2013, Langhammer* et al.* 2014). Since the two lines have been bred independently, they developed distinct biodiverse phenotypes. Although the two lines show similar litter sizes at first delivery, they differ in many physiological, behavioural and endocrine aspects (Langhammer* et al.* 2014, Michaelis* et al.* 2020). In addition, gene expression patterns in the testis differ among FL1, FL2 and an unselected control line (Michaelis* et al.* 2017, 2018). In a two-factorial breeding experiment, we recently described that the improved fertility phenotype mainly depends on the female genotype (Langhammer* et al.* 2017).

The estimation of reproductive parameters (e.g. litter size or mating rate) in mouse lines often refers to the values at the first delivery. However, litter size at first delivery might not be the most interesting fertility parameter in economically important farm animal species (e.g. pigs, sheep or rabbits) for which FLs might serve as bona fide animal models. For these species, increasing lifetime fecundity, including longevity, is more important and economically desired and ensures sustainable livestock farming. For various pig breeds, it has been described that litter sizes increase from litter to litter, reaching maximal levels at the 3rd to 4th delivery (Andreas* et al.* 2018). Therefore, we were interested in studying the lifetime fecundities of different mouse lines. We compared the FLs to an unselected control line (ctrl) that originated from the same initial founder population that was maintained without any selection pressure. We also included two additional Dummerstorf outbred mouse lines that were not selected for fertility traits. Line DU6 was selected for high male body weight at day 42, and line DU6P was selected for high protein mass at day 42 (Bunger* et al.* 1998, Renne* et al.* 2013). The aim of the present study was to compare two mouse lines selected for high fertility (FLs) to a control fertile line (ctrl) and two low-fertility lines (DU6 and DU6P). We focused on fertility parameters at first delivery and addressed questions of lifetime fecundity and longevity.

## Materials and methods

### Animals

All procedures were performed in accordance with national/international guidelines and were approved by the state of Mecklenburg-Western Pomerania and the Animal Protection Board from the Leibniz Institute for Farm Animal Biology. The animals were maintained in a specific pathogen-free (SPF) environment with defined hygienic conditions at a temperature of 22.5°C, at least 45% humidity and a controlled light regime with a 12 h light:12 h darkness cycle. The mice were kept in polysulfone cages of 267 × 207 × 140 mm (H-Temp PSU, Type II, Eurostandard, Tecniplast, Germany) or type II long cages (365 x 207 x 140 mm) for the larger line of animals and had free access to pellet concentrate and water. A standard breeding diet with 22% crude protein, 34% starch, 5% sugar, 4.5% crude fat, 3.9% crude fibre, 50.1% N free extracts and a 3.2% mineral mixture (ssniff M-Z autoclavable, Soest, Germany) was provided* ad libitum*.

FL1 and FL2 lines were selected by the Dummerstorf fecundity index, which was calculated as fecundity index = 1.6 × litter size at birth + total litter birth weight (g) in primiparous females. Beginning with generations 174 (FL2) and 175 (FL1), selections were performed by BLUP (best linear unbiased prediction) breeding value estimation, focusing only on the number of pups in primiparous females.

In 1975, selection on growth was started, creating the Dummerstorf growth line DU6 by selection for high body mass by sib/family selection (Renne* et al.* 2003). In every generation, 80 paired matings were made at an age of 63 ± 3 days (Bunger* et al.* 1998). After 169 generations of selection body mass, the 6-week male body weight increased from 29.8 to 83.2 g.

The line DU6P was selected for total protein mass in the male carcass at day 42 under conventional housing up to generation number 151 (Bunger* et al.* 1998). During the selection period, protein mass increased from 3 to 6.8 g.

Starting in 2014, the increasing inbreeding coefficient in the selection lines was controlled by the ‘Optimal Genetic Contributions to the Next Generations’ method of Meuwissen (1997).

### Determination of the productive mating rate and litter size at first parturition

All FBN selection mouse lines were kept with population size of 60 breeding pairs. The control line is maintained in a population size of 125 breeding pairs. To describe their momentary status regarding the fertility traits ‘litter size at first parturition’ (equal to number of live-born pups per litter) and ‘productive mating rate’ (equal to pregnancy rate within a 2-week breeding period), we calculated the average of these characteristics over the last 22 generations in each of the mouse lines.

### Design of the lifetime fecundity experiment

Animals were randomly selected from the five purebred lines after the first successful delivery in the course of the standard breeding protocol. In summary, we used 30 females of DU6 and DU6P, 50 females of the ctrl and FL1 lines and 60 females of the fertility line FL2. The included dams from the ctrl, FL1 and FL2 lines came from two different successive groups, which was considered in the statistical model. Females were mated in a 1:1 female/male ratio with an adult buck from the same line. In Supplementary Fig. 1 (see section on supplementary materials given at the end of this article), we provide details on the rotation protocol of bucks. Before being placed in a cage with the male, the dams were weighed at each round of delivery. Male and female animals were caged for 2 weeks, males were removed from the cage, and females were maintained for an additional 3-week observation period to give birth. If the female gave birth to offspring, we counted this as 'delivery in the first round'. If the female did not conceive, she was placed with another buck (Supplementary Fig. 1). Again, males and females were caged together for 2 weeks followed by a 3-week observation period. If the female gave birth to offspring in the second round, we counted this as 'delivery in the second round'. If the female failed to deliver in the first and second rounds, she was removed from the experiment due to impaired fertility. If the female delivered in the first or second round, standardization of litter size was performed immediately after birth to ten (five males and five females) newborn pups, and the offspring were suckled until weaning at 21 days of age. To account for losses during the suckling period, we standardized all litters to ten pups for comparisons between lines. Thus, only litters with at least ten pups per litter were included in this analysis. In cases where fewer than ten were born but another pup of the same line was born on the same day, we added pups to increase the litter size to ten. Directly after weaning, females re-entered the mating protocol. The time between the start of mating and birth of newborn pups was recorded to describe the length of the gestation period for each litter. Animals were removed from the experiment if they did not deliver or if they required a medical examination of their general health status. All included animals were observed daily to assess their health. If physical impairments due to inflammation, tumours or intestinal excrescence were detected that would cause suffering or substantial pain to the animals, we culled those females and documented their age and reason for removal. Otherwise, the age of death was noted if the animals died of natural causes.

### Statistical analysis

The data analysis was performed using R 3.5.2 (R Core Team 2018) and SAS software (Version 9.4 for Windows, SAS Institute Inc., Cary, NC, USA). Descriptive statistics and tests for normality were calculated with the UNIVARIATE procedure of Base SAS software (SAS Institute Inc. 2013. Base SAS® 9.4 Procedures Guide, Second Edition. Cary, NC: SAS Institute, Inc.). The litter size at birth count data was analysed with the GLIMMIX procedure of SAS/STAT software (SAS Institute Inc. 2013. SAS/STAT® 13.1 User’s Guide, Cary, NC: SAS Institute Inc.) using a Poisson model with the fixed effects line (levels: CTRL, DU6, DU6P, FL1, FL2) and litter number (levels: 1, 2,…) within each line and group. The length of the reproduction period per dam was analysed only as the remaining time in the experiment in days. For the analysis of the duration of the reproduction period per dam, we used the LIFETEST procedure of SAS/STAT software to compute nonparametric Kaplan–Meier (product-limit) estimates of the survivor functions for the five lines to compare the survival curves of these lines. In addition, least-squares means (LSMeans) and their s.e. were computed for each level of the fixed effects in the models, and all-pairwise differences of LSMeans were tested by the Tukey–Kramer procedure. Tukey’s all-pairwise comparison of litter size per line was calculated by the function glht from the multicomp package (V 1.4-14) (Hothorn* et al.* 2008). Effects and differences were considered significant if *P*  < 0.05.

## Results

### Mouse models of high, medium and low fertility at first delivery

**Figure 1:**
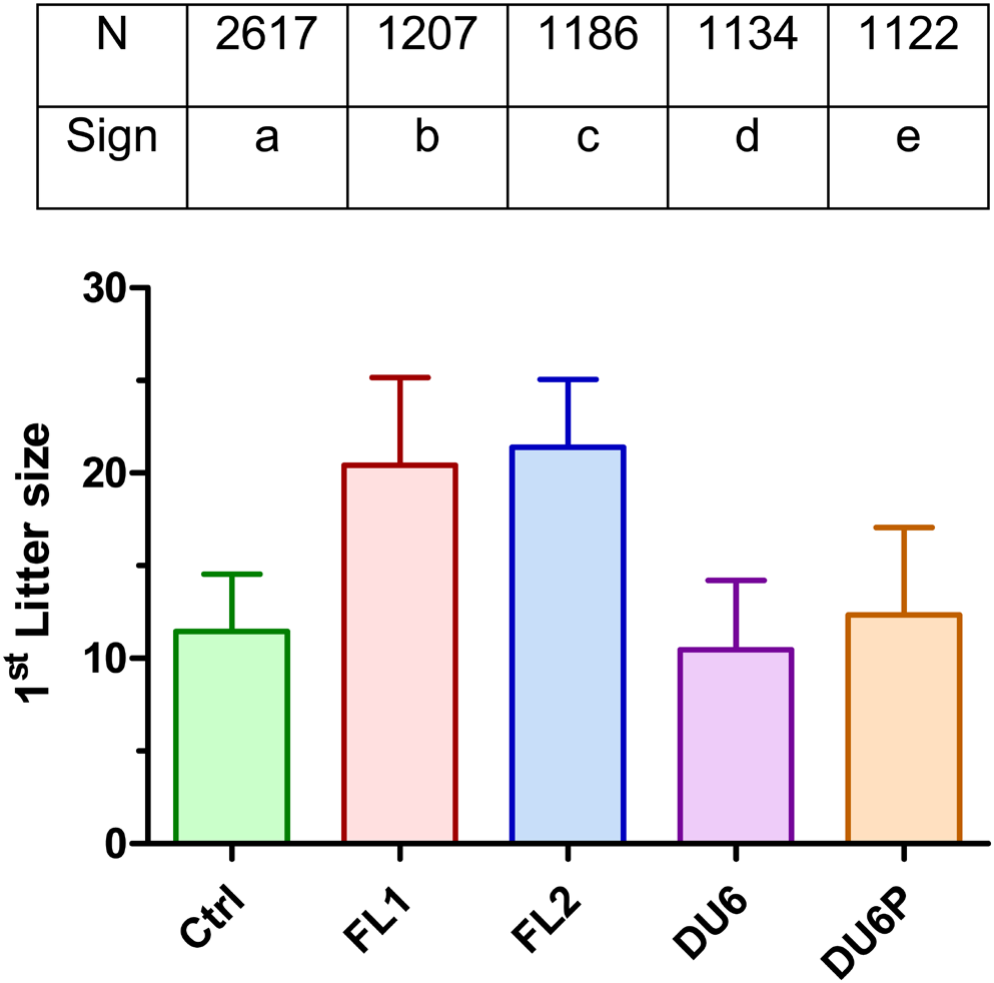
Litter sizes at the first delivery of five Dummerstorf outbred mouse lines. Male and female animals of the indicated lines were mated together in a 1:1 ratio for 2 weeks. Data are summarized for 22 generations with 60–125 breeding pairs per generation (Mean ± s.d.). N indicates the number of investigated breeding pairs, and different letters indicate statistically significant differences (sign). The means were tested by Tukey’s all-pairwise comparison (*P* < 0.05). Ctrl, unselected control line; FL1, fertility line 1; FL2, fertility line 2; DU6, high body weight line; DU6P, high protein line.

We previously described two outbred mouse lines that were selected for high fertility via breeding for more than 45 years and 174 generations. The actual status over the last 22 generations of selection regarding the fertility traits ‘litter size at first parturition’ and the ‘percentage of productive mating’ are displayed in Fig. 1 and Table 1. During the long breeding period for high body mass, it became obvious that high-growth line DU6 developed reduced fertility that was not intended and might be a side effect of selection for high body weight. Thus, this line was included in the experiment as an example of a line with low fertility. Fertility lines FL1 and FL2 showed comparable high productive mating rates (90–91%) as that of the ctrl line (96%). However, the rate of productive mating was slightly reduced in the DU6P high-protein line (86%). A significantly reduced mating performance was especially noticed in the heavyweight DU6 mouse line (63%) (Table 1). In contrast to the productive mating rates, the mean litter sizes at first delivery appeared to be unaffected in ctrl (11.5), DU6 (10.4) and DU6P (12.3), whereas litter sizes were elevated in FL1 (20.6) and FL2 (21.4) (Fig. 1).

**Table 1:** Productive mating rates of five Dummerstorf mouse lines.

Line	Mean, %
Ctrl	95.6
FL1	91.0
FL2	90.1
DU6	63.4
DU6P	86.4

Productive mating rates (given as a percentage) over 22 generations with 60–125 breeding pairs per generation for each line. Productive mating rates were determined based on delivered offspring after a 1:1 male/female mating period of 2 weeks.

Ctrl, unselected control line; FL1, fertility line 1; FL2, fertility line 2; DU6, high body weight line; DU6P, high protein line.

### No obvious health problems were present in the high-fertility mouse lines

**Figure 2:**
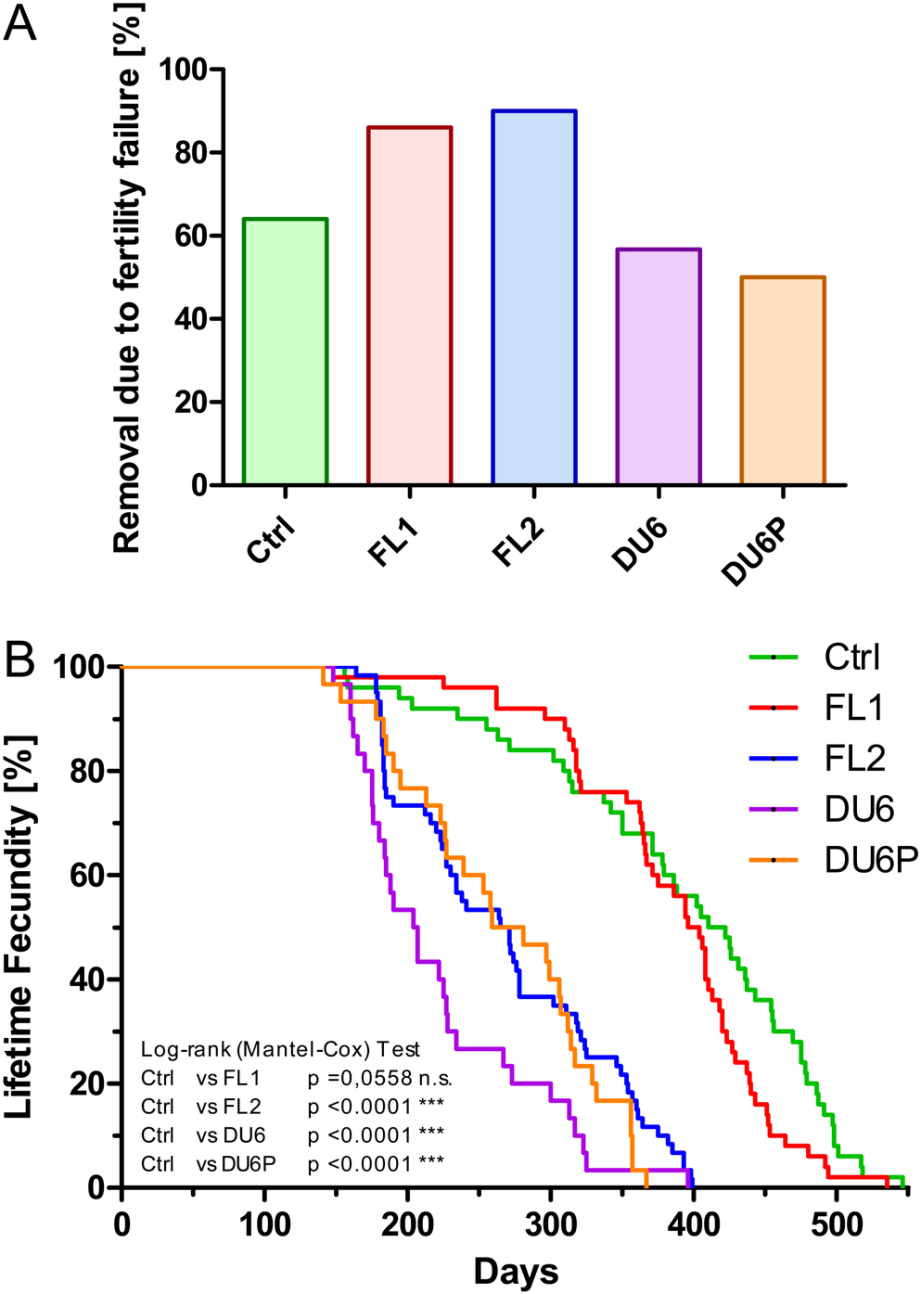
(A) Retirement due to fertility failure. Retirement (given as a percentage) of animals that had to be removed from the experiments due to fertility problems. Males and females were caged in a 1:1 ratio for 2 weeks, followed by a 3-week observation window. In cases of unsuccessful delivery, this mating protocol was repeated. Animals that had to be removed from the experiment due to fertility problems are indicated. Alternatively, animals had to be removed from the experiment due to various health problems. Ctrl, unselected control line; FL1/FL2, fertility lines 1 and 2; DU6, high body weight line; DU6P, high protein line. (B) Lifetime fecundity of five Dummerstorf mouse lines. Females had to be removed from the experiment either due to inappropriate fertility or to health problems. The number of days for which the females remained in the experiment is shown for five Dummerstorf mouse lines as a Kaplan–Meier plot. Ctrl, unselected control line; FL1/FL2, fertility lines 1 and 2; DU6, high body weight line; DU6P, high protein line.

As shown in Fig. 2A, between 85 and 90% of females of the two high-fertility lines FL1 and FL2 were removed from the lifetime fecundity experiment due to impaired fertility, meaning that only 10–15% of these females were removed from the experiment due to health reasons or because of natural death. This portion was considerably higher in the other lines (up to 50% in DU6P, Fig. 2A).

To correct these data, we analysed at which age the animals had to be removed from the experiment independent of the failure criterion. We observed that DU6, DU6P and FL2 females had to be removed from the experiment after 223, 267 and 269 days (on average, age counted from birth), whereas females of FL1 and ctrl remained in the breeding experiment for 385 and 393 days, respectively (Fig. 2B). In addition, we analysed these data by splitting the females into subgroups that had to be removed from the experiment for fertility or other health reasons; however, we did not obtain significantly different results compared to the combined analysis shown in Fig. 2B (data not shown).

Furthermore, we observed several line-specific differences according to fertility and other physiological parameters. Females of the DU6 line require a longer period for gestation, especially in later matings (Supplementary Fig. 2) and were considerably heavier than females of the other lines (Supplementary Fig. 3).

### Number of offspring and number of litters per lifetime

**Figure 3:**
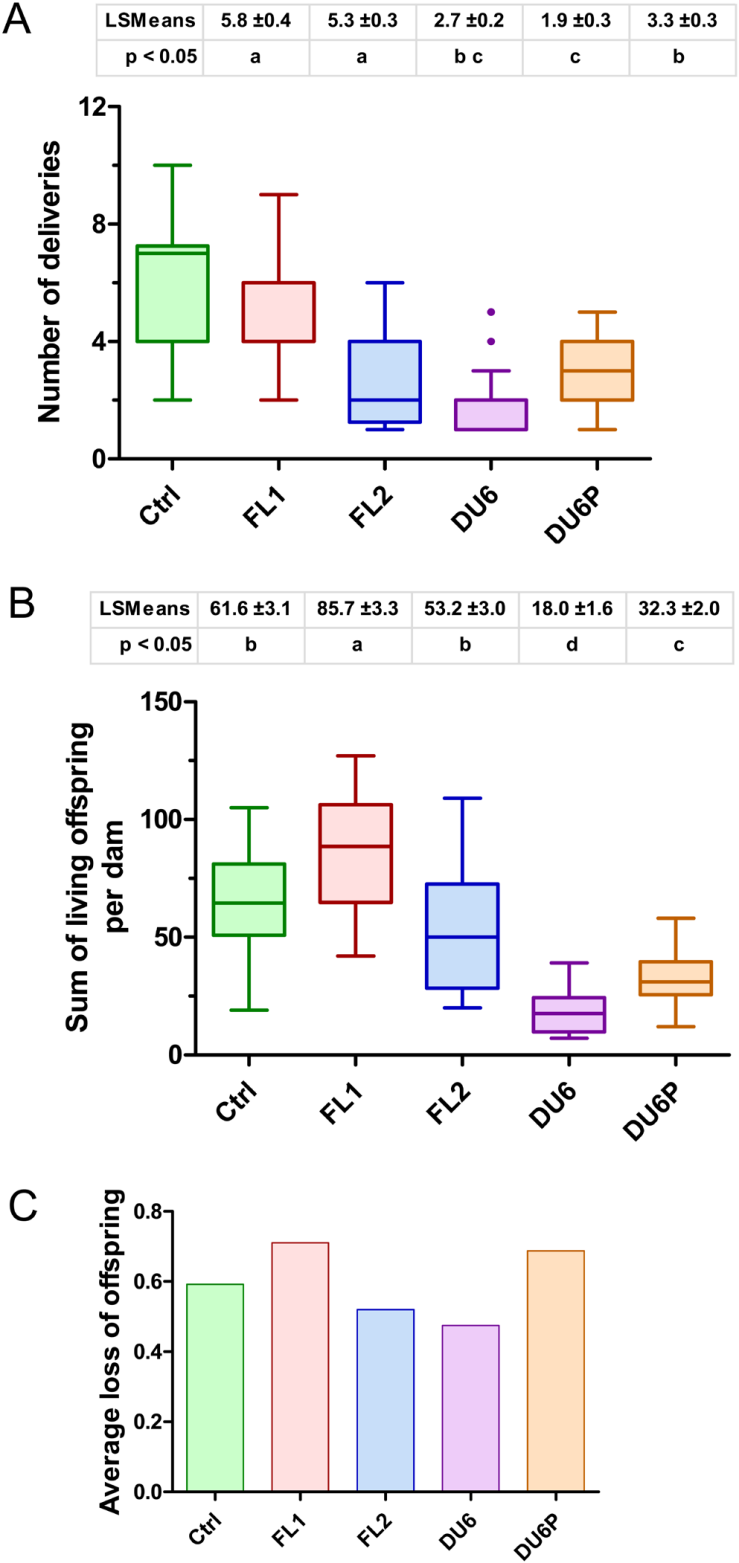
(A) Lifetime fecundity: number of deliveries per dam. The number of deliveries per dam (LSMeans ± s.e. ) and its distribution (boxplot) are shown for five Dummerstorf mouse lines. Males and females were caged in a 1:1 ratio for 2 weeks, followed by a 3-week observation window. In cases of unsuccessful delivery, this mating protocol was repeated. If the animals did not deliver offspring in the first and second matings, they were removed from the experiment due to fertility problems. Ctrl, unselected control line; FL1/FL2, fertility lines 1 and 2; DU6, high body weight line; DU6P, high protein line. LSMeans were tested by the Tukey–Kramer procedure. Different letters indicate statistically significant differences (*P*  < 0.05). (B) Lifetime fecundity: number of live-born offspring per dam. The number of live-born offspring per dam (LSMeans ± s.e. ) and its distribution (boxplot) of five Dummerstorf mouse lines are given. LSMeans were tested by the Tukey–Kramer procedure. Different letters indicate statistically significant differences (*P*  < 0.05). Ctrl, unselected control line; FL1/FL2, fertility lines 1 and 2; DU6, high body weight line; DU6P, high protein line. (C) Number of losses during the suckling period up to weaning at 3 weeks of age. Males and females were caged in a 1:1 ratio for 2 weeks, followed by a 3-week observation window. In cases of unsuccessful delivery, this mating protocol was repeated. Litter sizes were standardized to ten pups per litter, and the losses of offspring between birth until weaning at 21 days of age are indicated. Ctrl, unselected control line; FL1/FL2, fertility lines 1 and 2; DU6, high body weight line; DU6P, high protein line.

In the next experiment, we addressed the question of how many litters each dam could deliver. Here, we observed large differences between the different lines. Whereas DU6 females had an average of only 1.9 litters/animal, females of FL1 and ctrl had averages of 5.3 and 5.8 litters per animal, respectively (Fig. 3A).

These differences in fertility are partly reflected by the total number of offspring per lifetime and per female. On average, females of DU6 gave birth to 18.0 ± 1.6 offspring per lifetime, whereas females of FL2, ctrl and FL1 delivered 53.2 ± 3.0, 61.6 ± 3.1 and 85.7 ± 3.3 offspring per lifetime, respectively (Fig. 3B). In this analysis, we counted living pups per dam. When every offspring (sum of live- and dead-born pups) was included in the analysis, we did not observe different patterns between lines compared to just counting live-born offspring (Supplementary Fig. 4). In line with this observation, there were no significant differences between the mouse lines regarding the average number of pups lost during the suckling period up to weaning at 3 weeks of age (between 0.5 and 0.7; Fig. 3C).

### Litter size and pregnancy rate per litter

**Figure 4:**
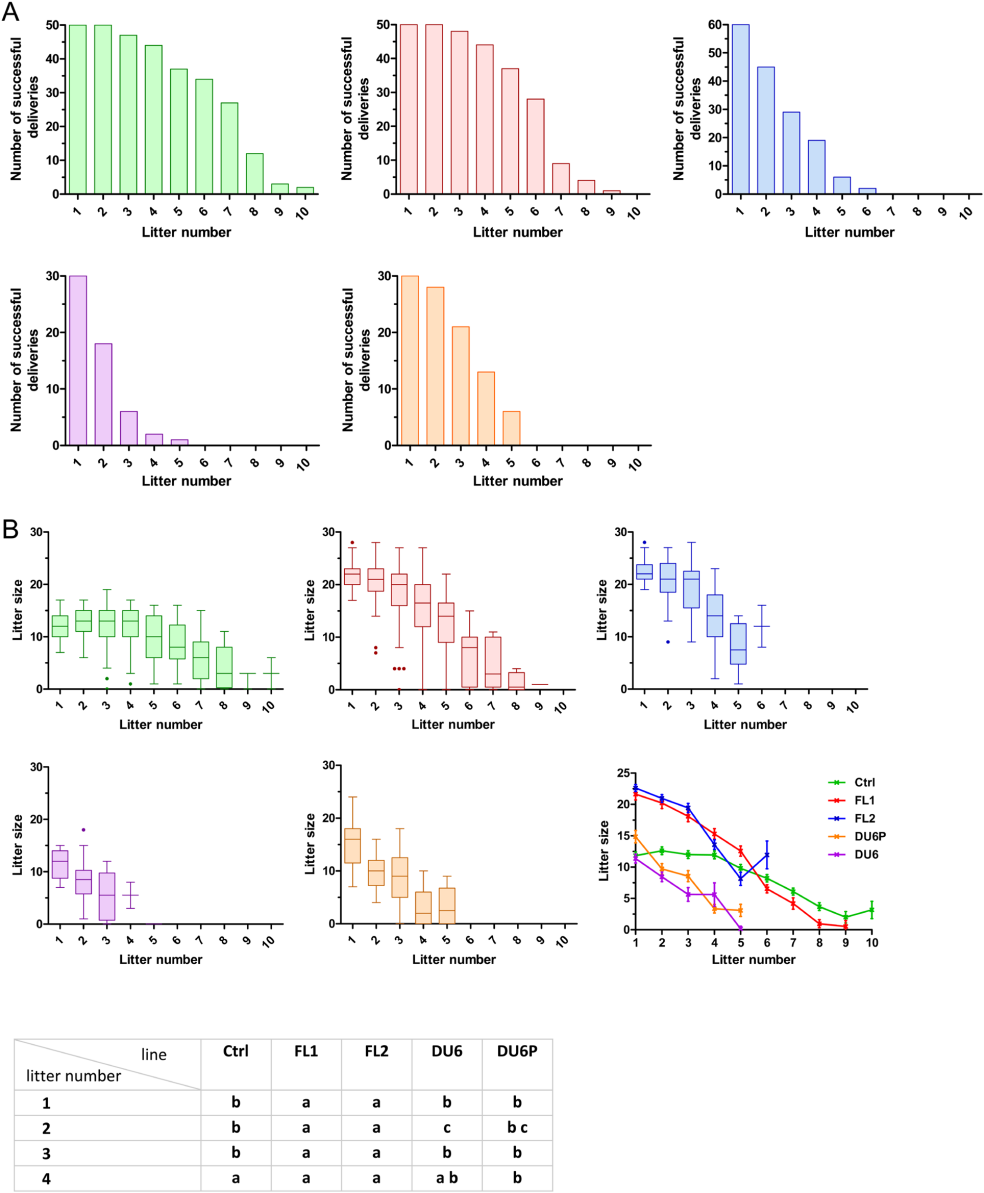
(A) Number of animals remaining in the experiment per litter. Males and females were caged in a 1:1 ratio for 2 weeks, followed by a 3-week observation window. In cases of unsuccessful delivery, this mating protocol was repeated. If the animals did not deliver offspring following the first and second matings, they were removed from the experiment due to fertility problems. Alternatively, animals could be removed from the experiment due to general health problems. Ctrl, unselected control line (green); FL1, fertility line 1 (red); FL2, fertility line 2 (blue); DU6, high body weight line (purple); DU6P, high protein line (orange). (B) Litter size per litters. The number of offspring per litter number (LSMeans ± s.e. ) and its distribution (boxplot) are shown for five Dummerstorf mouse lines. LSMeans were tested between lines and within the first four litters by the Tukey–Kramer procedure. Different letters indicate statistically significant differences (*P*  < 0.05). Ctrl, unselected control line (green); FL1, fertility line 1 (red); FL2, fertility line 2 (blue); DU6, high body weight line (purple); DU6P, high protein line (orange).

In the next analysis, the question of how many animals of a particular line remained in the experiment at each round of mating was addressed. The number of dams able to produce subsequent litters after their first litter provides an indication of the female lifetime fertility for each line. As shown in Fig. 4A, none of the females of DU6 and DU6P delivered more than six litters, whereas 56% and 68% of FL1 and ctrl females successfully produced a sixth litter (Fig. 4A). Females of the FL1 and ctrl lines delivered up to nine and ten litters, respectively (Fig. 4A and B). Very similar declines were observed in the litter sizes for each line, which was again most pronounced in lines DU6 and DU6P. One remarkable exception was the ctrl line, in which litter capacity remained constant during the first four deliveries (Fig. 4B).

In this experiment, we also counted the average litter size per female within the first four litters to eliminate the negative effect of declining litter sizes as the number of matings increased for the ctrl and FL1 lines. We observed the highest litter sizes per female in FL2 (20.4 offspring/female), with slightly lower litter sizes in FL1 (18.9 offspring/female) and the other lines (DU6: 9.6, DU6P: 10.3 and ctrl: 12.1) (Table 2A). Although the litter sizes of FL1 and FL2 were almost doubled compared to the control line, the average birth weight of the newborn pups was not lower (Table 2B).

**Table 2:** Mean number of offspring and mean average pup birth weight.

Line	*n*	Min	Max	Mean	s.d.	*P* < .05
A: Average litter size						
Ctrl	191	0	19	12.12	3.32	c
FL1	192	0	28	18.92	5.46	b
FL2	153	2	28	20.41	4.67	a
DU6	56	0	18	9.64	4.00	d
DU6P	92	0	24	10.33	5.60	d
B: Average mean pup birth weight						
Ctrl	190	1.40	2.72	1.84	0.19	b
FL1	190	1.38	2.58	1.87	0.19	b
FL2	153	1.64	2.32	1.88	0.13	b
DU6	55	2.07	3.63	2.63	0.28	a
DU6P	86	1.79	3.42	2.36	0.28	a

Different letters indicate statistically significant differences (*P* < .05).

The average litter size (A) and the average mean pup birth weight (B) of the first four litters are shown for five Dummerstorf mouse lines. Males and females were caged in a 1:1 ratio for 2 weeks, followed by a 3-week observation window. In cases of unsuccessful delivery, this mating protocol was repeated. The means were tested by Tukey’s all-pairwise comparison by the multicomp package of R.

### Percentage of litters delivered from the second mating

**Figure 5:**
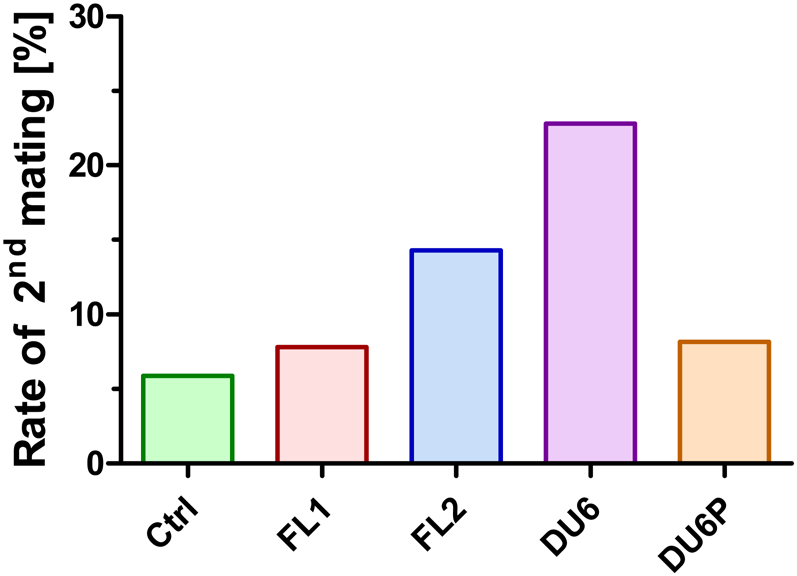
Percentage of litters derived from the second mating. Males and females were caged in a 1:1 ratio for 2 weeks, followed by a 3-week observation window. In cases of unsuccessful delivery, this mating protocol was repeated. The percentage of litters born after a second mating which allowed the dam to meet the criteria and to remain in the experiment is indicated. Ctrl, unselected control line; FL1/FL2, fertility lines 1 and 2; DU6, high body weight line; DU6P, high protein line.

Since the productive mating rates in the purebred lines already indicate substantial differences (Table 1), we analysed whether females of the different lines needed a second mating to become pregnant and to remain in the experiment (see breeding protocol, above). We observed that 23% of DU6 and 14% of FL2 litters were born after the second round of mating, whereas these rates were much lower for the other selection lines (Fig. 5). This indicates that DU6 (and in part FL2) has problems conceiving, especially in later mating attempts (Supplementary Fig. 2).

### Fecundity index

To further compare the fertility characteristics of the investigated mouse lines, we integrated three fertility parameters into a comprehensive fecundity index according to Silver (1995). This index integrates (i) the litter size (at first delivery), (ii) numbers of litters born per dam (lifetime fecundity) and (iii) the productive mating rate (at first delivery). The highest fecundity rates were observed for FL1 with >100 index units (Table 3). We also included data from the QSi5 line, which has been selected for high fertility. Additionally, we included fertility data of commonly used inbred lines (such as C57BL/6J and BALB/cJ), which show reduced fertility (Table 3).

**Table 3:** Silver fecundity index.

Strain	Litter size	Litters per dam	Productive mating (%)	Fecundity index	Reference
Ctrl, Dummerstorf	11.5	6.1	95.6	67.1	Present manuscript
FL1, Dummerstorf	20.6	5.4	91.0	101.2	Present manuscript
FL2, Dummerstorf	21.4	2.7	90.1	52.1	Present manuscript
DU6, Dummerstorf	10.4	1.9	63.4	12.5	Present manuscript
DU6P, Dummerstorf	12.3	3.3	86.4	35.1	Present manuscript
C57BL/6J	7.0	4.0	84.0	23.5	Silver (1995)
BALB/cJ	5.2	3.8	47.0	9.3	Silver (1995)
QSi5	13.4	5.0	97.0	65.0	Wei *et al.* (2013)

Estimation of the fecundity index according to Silver, the Silver fecundity index integrates (i) the litter size (at first delivery), (ii) numbers of litters born per dam (lifetime fecundity) and (iii) the productive mating rate (at first delivery).

## Discussion

We previously described two highly fertile mouse lines (FL1 and FL2). Based on selection over >45 years and 190 generations, these mouse lines almost doubled the number of offspring compared to a control line. From the very beginning of the selection process, both lines were developed separately and are now highly diverged mouse lines showing various differences in their physiological, behavioural, endocrine and molecular characteristics (Langhammer* et al.* 2014, Michaelis* et al.* 2013, 2020). Nevertheless, both biodiverse mouse lines produce double the number of offspring, namely, 20.6 and 21.4 pups/litter (Fig. 1) and have double the number of ovulated oocytes per cycle (Spitschak* et al.* 2007). The selection criteria were originally based on a mixed index including the number of offspring and the total birth weight. Both parameters were determined at the first delivery and might not reflect the lifetime fecundity of the animals. To obtain a more complete picture, we tested these two highly fertile mouse lines together with the unselected control line and two additional Dummerstorf long-term selection lines that were selected for high body weight and high protein mass without selection on fertility parameters. These lines responded differently regarding fertility parameters during long-term breeding in a lifetime fecundity study.

Unfortunately, no standard and generally accepted protocol exist for determining lifetime fecundity in mice. Most recommendations are rather vague, for example, recommending retiring breeding pairs 'when the caretaker judged that the period of optimal breeding performance had ended for that breeding pair, based on experience and general guidelines of the strain' (The Jackson Laboratory Handbook, www.phenome.jax.org). Most recommendations include the following suggestions: (i) replace females after 60 days without producing a litter and/or (ii) replace females if the female produces pups but the pups do not survive for two to three litters (The Jackson Laboratory Handbook, www.phenome.jax.org). Based on these basic parameters, we set up the present experimental design. The protocol should include suckling of offspring to achieve a more comprehensive picture of the fertility of the mouse lines. Thus, females were mated together with a buck from the same line for 2 weeks followed by a 3-week observation period to determine whether the female conceived. In the case of an unsuccessful mating, the 2-week mating period followed by a 3-week observation period was repeated. Only those females that did not deliver offspring in this dual mating protocol were removed from the experiment due to impaired fertility. As mating partners, we selected adult bucks from the same line that were replaced after three to four rounds of mating. This protocol minimizes the impact of one particular buck since we recently described that the reproductive performance in Dummerstorf high-fertility mouse lines primarily depends on the female genotype in a two-factorial breeding experiment (Langhammer* et al.* 2017).

Litter sizes at first delivery measured within the 22-generation observation window presented in the current manuscript (Fig. 1) are identical to those reported in the earlier work (Michaelis* et al.* 2017, 2018). This was expected since the first deliveries have been previously compared. However, from the second litter, the litter sizes started to decline, and the number of animals remaining in the experiment also declined. This decline was most pronounced in DU6 and DU6P, which are selected for high body weight and high protein mass (Fig. 4A and B). None of the DU6 or DU6P females delivered a sixth litter. Additionally, the litter sizes of the fifth (DU6) and fourth and fifth (DU6P) litters dropped from zero to three pups per litter (Fig. 4B), clearly indicating that older DU6 and DU6P dams experience decreased fertility but not at first delivery. They do not deliver a large number of pups overall, and the litter sizes decline rapidly.

Additionally, FL2 females had a limited number of mating attempts (a maximum of up to six litters); however, at least in the first three litters, the litter sizes were very large (Fig. 4B). This is in line with the observation that FL2 females needed a second attempt at mating more frequently than FL1 and ctrl females (Fig. 5). Consequently, the fertility phenotype of FL2 led to very high average litter sizes within the first four matings compared to the other lines (Table 2). This phenotype partly resembles the phenotype of FSH-overexpressing mice. These mice initially have higher litter sizes than WT littermates; however, these litter sizes rapidly decline in later rounds of breeding, leading to reduced lifetime fecundity compared to control animals (McTavish* et al.* 2007). Another example of a line with elevated litter sizes is Lin28-overexpressing mice (Zhu* et al.* 2010). However, since elevated expression of Lin28 is associated with ovarian cancer with poor prognosis (Peng* et al.* 2010), overexpression of Lin28 in the ovaries could interfere with long-term fertility. In contrast to Lin28-overexpressing mice, we did not observe any health problems in FL2 females (see below).

Females of FL1 and ctrl could deliver up to nine and ten litters (Fig. 4A and B). In contrast to the other lines, the litter sizes of the ctrl remained constant for the first four deliveries. This phenome or even a slight increase has been described for other mouse lines (Taketo* et al.* 1991), and a significant increase in litter size has been reported for other polytocous species, such as pigs (Andreas* et al.* 2018). For pig breeding, this aspect is highly desirable since these animals reach their maximum breeding potential and, therefore, their maximum economic value at their 3rd–4th delivery. Another aspect that is highly desirable in pigs is a high lifetime fecundity. Regarding this point, it should also be noted that FL1 and ctrl remained in the experiment for much longer time spans (Fig. 2B) than the other lines. For example, for mating #6, 56% and 68% of FL1 and ctrl females, respectively, were still in the experiment, whereas none of the DU6 or DU6P animals remained (Fig. 4A and B). Thus, the high-fertility phenotype of FL1, which combines large litter sizes with high lifetime fecundity, might be an interesting breeding goal for pig breeders compared to an FL2-like fertility phenotype, which combines initially large litter size with a reduced lifetime fecundity.

From the presented data, we cannot conclude that the frequent delivery of large litter sizes causes any health problems. The postpartum losses of newborns were not increased in the high-fertility lines compared to the other lines if the number of suckling pups was standardized to 10 immediately after birth (Fig. 3C). In this context, it should be acknowledged that losses during the suckling period are greatly increased if dams have to suckle unstandardized litters. If we did not standardize litter sizes to ten and females had to suckle their entire litter (independent of the litter size), embryonic losses increased to 28.2% (FL1) and 43.3% (FL2) (unpublished data). However, we would like to point out that the corresponding animal material is fundamentally different between the present study and the unstandardized data set mentioned above. These animals were from generations 146–162 (FL2) and 148–164 (FL1) from 2007 to 2011 with different litter sizes at that time and from our previous mouse facility without SPF standards. Nevertheless, it is tempting to speculate that postnatal losses might increase if litter sizes are not standardized. In contrast, the individual birth weight of FL offspring was not different from that of offspring from the control line even if litter sizes were high (Table 2B), probably because this criterion was part of the selection index. Based on these data, we roughly calculated that litter sizes between ten and fifteen would be an optimal value to deliver numerous healthy offspring.

Furthermore, for highly fertile dams, there was no increased incidence of being removed from the experiment due to health problems (Fig. 2A). Moreover, they seem healthier than females from the other lines.

Combining all these data together in a fecundity index that includes (i) litter size, (ii) litters born per dam, and (iii) the productive mating rate (‘Silver fecundity index’ (Silver 1995)), it became obvious that FL1 had by far the highest fecundity index of the studied lines. These mice delivered large litters with a high mating rate and over a long period, with a fecundity index of 101. The fecundity index of FL2 was much lower (52, Table 3) since they delivered very large numbers of offspring but at a low frequency. Since ctrl animals reached a Silver fecundity index of 67, FL2 might not be considered highly fertile according to this index. However, it should be noted that the QSi5 line, which originates from a mouse line that was selected for very large litter sizes at first delivery (Wei* et al.* 2013), did not show an elevated Silver fecundity index (65, Table 3) compared to ctrl. DU6 and DU6P showed reduced fecundity indices of 13 and 35, respectively. In this context, it should be noted that commonly used (wild-type) inbred mouse lines, such as C57BL/6J and BALB/cJ, show low fecundity indices of 24 and 9 and thus should be considered subfertile.

In addition to the ‘Silver fecundity index’, one could also apply other fecundity indices, for example, the recently described ‘simplified method to measure mouse fertility’ (Handelsman* et al.* 2020). This index summarizes the cumulative number of pups generated by repetitive mating without weaning within the first four litters. According to this measure (although both mating protocols are not entirely comparable), we would expect an outcome similar to what is shown in Table 2 for the five Dummerstorf outbred mouse lines. Thus, FL1 and FL2 would be the mouse lines with the highest fertility according to this index.

In view of the two high-fertility mouse lines FL1 and FL2 (high-fertility selection criterion: litter size of first delivery), it should be emphasized that they have alternative strategies to achieve their ‘high fertility’ phenotype. We have already noted differences in many physiological, behavioural, endocrine and molecular aspects in previous analyses, including a prolonged life expectancy in FL1 (Langhammer* et al.* 2014). In view of lifetime fecundity, we also see dramatic differences between FL1 and FL2. Both lines can deliver very large litters at first delivery; however, in comparison to FL1, the capacity of FL2 to repetitively deliver offspring is much more restricted. Thus, we present here two animal models with high fertility at first delivery but with differing long-term reproductive fitness. This aspect might be highly interesting to support the aim of sustainable agriculture. A high-fertility phenotype that combines a very large litter size with a high lifetime fecundity (FL1-like phenotype), might be highly preferable compared to the FL2-like phenotype, which combines very large initial litter sizes with a reduced lifetime fecundity. Furthermore, selection for increased fertility seems to be beneficial for general health. In turn, it is tempting to speculate that we have primarily selected for good health by selection for high fertility. Since questions of longevity in farm animals are important for sustainable agriculture, this aspect should be analysed in future experiments.

## Supplementary Material

1: Rotation of bucks. This table indicates how often bucks of a given generation and mouse lines were used for a mating protocol and how often they deliver pups. For example, 54 bucks from the control line (generation 173) are used. From these 54 bucks, 23 were mated in one mating attempts, 25 in two and 6 in three mating attempts. Of these 54 bucks, 1 delivered zero litters with pups, 28 delivered one, 20 delivered two and 5 delivered three litters with pups. This table illustrates that bucks have been used randomly.

2: Time between mating and litter birth in days. Ctrl: unselected control line (green); FL1: fertility line 1 (red); FL2: fertility line 2 (blue); DU6: high body weight line (purple); DU6P: high protein line (orange).

3: Dams’ body mass at the time of mating. Ctrl: unselected control line; FL1/FL2: fertility lines 1 and 2; DU6: high body weight line; DU6P: high protein line (means ± SD).

4: Sum of total offspring per dam and lifetime. The average total number of pups born per dam (sum of living and stillborn pups) (LSMeans ± SE) and its distribution (boxplot) of five Dummerstorf mouse lines are shown. Ctrl: unselected control line; FL1/FL2: fertility lines 1 and 2; DU6: high body weight line; DU6P: high protein line. LSMeans were tested by the Tukey-Kramer procedure. Different letters indicate statistically significant differences (p < 0.05).

## Declaration of interest

The authors declare that there are no conflicts of interest that could be perceived as prejudicing the impartiality of the research reported.

## Funding

Grants from the German Research Foundation (DFG, MI 2098/3-1) and the Leibniz Association (SOS-FERT, K52/2017) partly supported the research.

## Author contribution statement

M L, E W, M M, J S, N R and J M W conceived the study. M L, E W and N R were responsible for animal breeding. M L, E W, and A T conducted the statistical evaluation. M L, E W, M M and J M W analysed and interpreted the results and drafted the manuscript. All authors critically read the manuscript and approved the final draft.
