# Supplementary material for: Two mouse lines selected for large litter size display different lifetime fecundities
Source: Reproduction. 2021 Apr 20;161(6):721–30. doi: 10.1530/REP-20-0563 (PMC8183634; doi:10.1530/REP-20-0563)
Supplement: 1: Rotation of bucks. This table indicates how often bucks of a given generation and mouse lines were used for a mating protocol and how often they deliver pups. For example, 54 bucks from the control line (generation 173) are used. From these 54 bucks, 23 were mated in one mating attempts, 25 in tw [file supplementary_figure_1.pdf]

Supplementary Figures

Supplementary Figure 1

			breedings/pairings					deliveries				
**Line**	**Generation**	**Bucks#**	**1**	**2**	**3**	**4**	**5**	**0**	**1**	**2**	**3**	**4**
**Ctrl**	173	54	23	25	6			1	28	20	5	
	174	31	3	24	4				7	21	3	
	175	31	2	6	18	4	1	4	4	14	7	2
	176	8		3	5			5	2	1		
	177	9	9					8	1			
	178	41	24	15	2			1	25	13	2	
	179	23	14	9				6	11	6		
	180	19	14	5				5	12	2		
	181	10	9	1				7	3			
	182	3	3					3				
**FL1**	173	40	10	10	20				11	14	15	
	174	32	4	27	1			1	9	21	1	
	175	29	4	20	5			6	15	7	1	
	176	23	19	4				20	3			
	177	33	14	10	9			3	13	11	6	
	178	24	10	14				5	12	7		
	179	22	15	7				9	11	2		
	180	11	7	3	1			9	2			
	182	2	2					2				
**FL2**	173	45	16	13	16			2	28	11	4	
	174	26	11	15				13	10	3		
	175	10	3	7				8	2			
	176	2	2					2				
	177	42	12	13	17			3	23	12	4	
	178	35	34	1				17	18			
	179	20	19	1				16	4			
	180	3	3					3				
**DU6**	177	48	23	12	10	3		13	23	9	3	
	178	19	13	6				13	6			
	179	6	6					5	1			
	180	1	1					1				
**DU6P**	177	54	26	22	6			3	32	16	3	
	178	32	20	10	2			10	20	2		
	179	18	18					17	1			

1: *Rotation of bucks.* This table indicates how often bucks of a given generation and mouse lines were used for a mating protocol and how often they deliver pups. For example, 54 bucks from the control line (generation 173) are used. From these 54 bucks, 23 were mated in one mating attempts, 25 in two and 6 in three mating attempts. Of these 54 bucks, 1 delivered zero litters with pups, 28 delivered one, 20 delivered two and 5 delivered three litters with pups. This table illustrates that bucks have been used randomly.
