# Supplementary material for: Two mouse lines selected for large litter size display different lifetime fecundities
Source: Reproduction. 2021 Apr 20;161(6):721–30. doi: 10.1530/REP-20-0563 (PMC8183634; doi:10.1530/REP-20-0563)
Supplement: 2: Time between mating and litter birth in days. Ctrl: unselected control line (green); FL1: fertility line 1 (red); FL2: fertility line 2 (blue); DU6: high body weight line (purple); DU6P: high protein line (orange). [file supplementary_figure_2.pdf]

Supplementary Figure 2

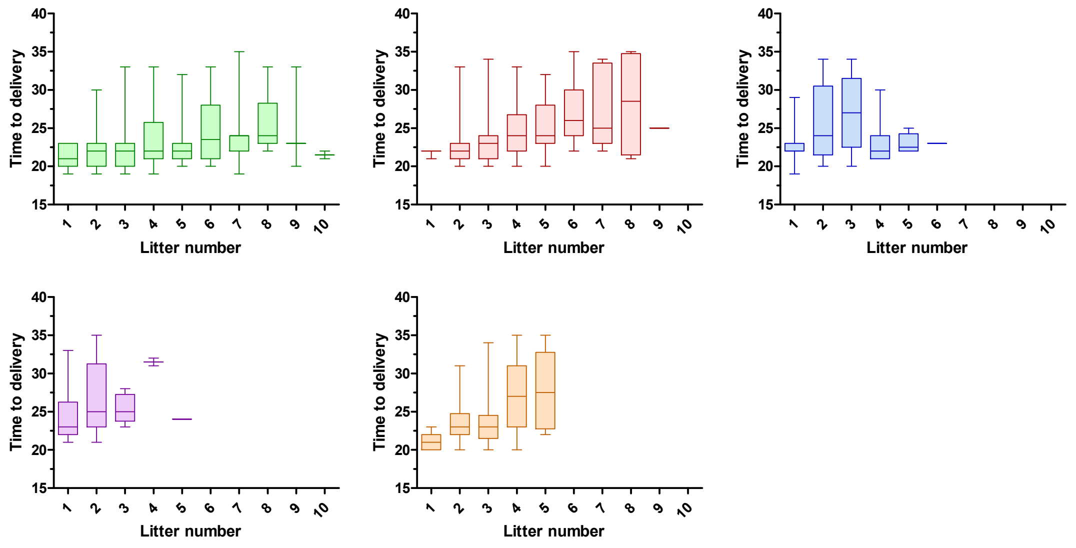

Time between mating and litter birth in days. Ctrl: unselected control line (green); FL1: fertility line 1 (red); FL2: fertility line 2 (blue); DU6: high body weight line (purple); DU6P: high protein line (orange).
