# Supplementary material for: Two mouse lines selected for large litter size display different lifetime fecundities
Source: Reproduction. 2021 Apr 20;161(6):721–30. doi: 10.1530/REP-20-0563 (PMC8183634; doi:10.1530/REP-20-0563)
Supplement: 3: Dams’ body mass at the time of mating. Ctrl: unselected control line; FL1/FL2: fertility lines 1 and 2; DU6: high body weight line; DU6P: high protein line (means ± SD). [file supplementary_figure_3.pdf]

Supplementary Figure 3

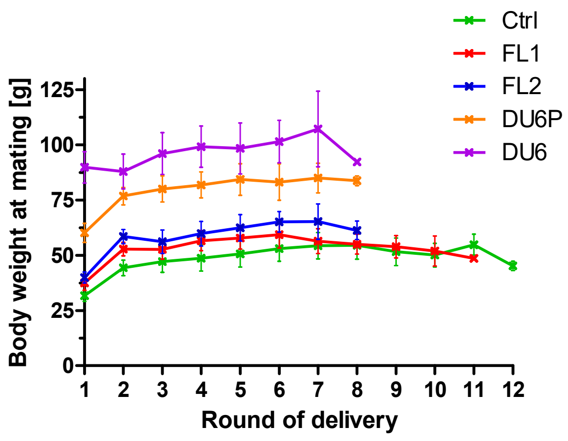

Dams' body mass at the time of mating. Ctrl: unselected control line; FL1/FL2: fertility lines 1 and 2; DU6: high body weight line; DU6P: high protein line (means ± SD).
