# Supplementary material for: Two mouse lines selected for large litter size display different lifetime fecundities
Source: Reproduction. 2021 Apr 20;161(6):721–30. doi: 10.1530/REP-20-0563 (PMC8183634; doi:10.1530/REP-20-0563)
Supplement: 4: Sum of total offspring per dam and lifetime. The average total number of pups born per dam (sum of living and stillborn pups) (LSMeans ± SE) and its distribution (boxplot) of five Dummerstorf mouse lines are shown. Ctrl: unselected control line; FL1/FL2: fertility lines 1 and 2; DU6: high body we [file supplementary_figure_4.pdf]

Supplementary Figure 4

LSMeans	63.0±3.1	89.9±3.1	53.7±3.1	19.7±1.7	34.8±2.3
p < 0.05	b	a	b	d	c

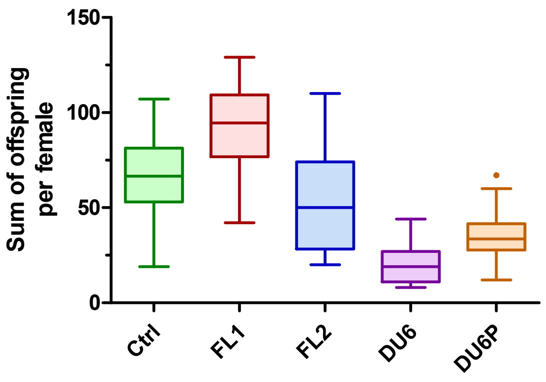

Sum of total offspring per dam and lifetime. The average total number of pups born per dam (sum of living and stillborn pups) (LSMeans ± SE) and its distribution (boxplot) of five Dummerstorf mouse lines are shown. Ctrl: unselected control line; FL1/FL2: fertility lines 1 and 2; DU6: high body weight line; DU6P: high protein line. LSMeans were tested by the Tukey-Kramer procedure. Different letters indicate statistically significant differences ($p < 0.05$).
